# Acute leg pain with suspected beginning leg compartment syndrome and deep vein thrombosis as differential diagnoses in an unusual presentation of Brodie’s abscess: a case report

**DOI:** 10.1186/s13256-015-0770-x

**Published:** 2015-12-23

**Authors:** Ahmed Hammad, Philip Johannes Felix Leute, Isabel Hoffmann, Sebastian Hoppe, Stefan Lakemeier, Hans-Michael Klinger

**Affiliations:** Abteilung für Orthopädie, Universitätsmedizin Göttingen, Robert-Koch-Strasse 40, 37075 Göttingen, Germany

**Keywords:** Acute leg pain, Brodie’s abscess, Deep vein thrombosis, leg compartment syndrome, Osteomyelitis

## Abstract

**Background:**

Brodie’s abscess is an uncommon form of subacute osteomyelitis where the main presenting symptom is mild to moderate pain of insidious onset for several months’ duration. We report a case of a patient presenting with acute leg pain resembling that of a deep vein thrombosis, and a beginning leg compartment syndrome following a suspected ruptured Baker’s cyst. Our case is unusual because of the acute presentation of the Brodie’s abscess with acute leg pain and acute swelling without any preceding trauma; to the best of our knowledge, this presentation has not been reported before.

**Case presentation:**

A 17-year-old white boy presented to our out-patient clinic with a 6-month history of pain in his left knee joint of insidious onset. There was no history of trauma to the extremity. After performing physical and radiological (X-ray) examinations, we initially diagnosed medial meniscus damage. One week later he presented to our emergency department with acute sudden increase in the pain and swelling of his left knee, and pain and swelling of his left leg, without any trauma. Deep vein thrombosis and beginning leg compartment syndrome from ruptured Baker’s cyst were initially diagnosed. Magnetic resonance imaging was performed and Brodie’s abscess was the most probable diagnosis. We performed open surgical debridement and curettage with drainage of the abscess and administered postoperative antibiotics. He presented to our out-patient clinic 3 months postoperatively, where he was pain-free with no residual local tenderness.

**Conclusions:**

In cases of sudden acute increase in joint or extremity pain or swelling that has been insidiously present for months, Brodie’s abscess should be considered as one of the differential diagnoses, as it may present acutely in cases with accompanying fasciitis and myositis and be clinically mistaken for deep vein thrombosis or limb compartment. Magnetic resonance imaging remains the gold standard imaging study, and surgical treatment followed by postoperative antibiotics remains the standard treatment.

## Background

Osteomyelitis is an inflammatory process accompanied by bone destruction, which is caused by an infecting microorganism [1, 2]. There are several ways to classify osteomyelitis. The two main classification systems are the Waldvogel classification system [2] and Cierny–Mader classification [3]. The Cierny–Mader classification is based on both the anatomy of the bone (medullar, superficial, localized, or diffuse) and three physiological classes (normal host, systemic and/or local compromise, treatment worse than the disease) [1, 3, 4]. The Waldvogel classification considers the duration of the disease (acute/chronic), the mechanism of infection (hematogenous/contiguous seeding) and the presence of vascular insufficiency [1, 2, 4]. Osteomyelitis is further subdivided into acute and chronic osteomyelitis according to the duration of the disease. Acute osteomyelitis evolves over several days or weeks [2] before osteonecrosis has occurred [1, 5]. Chronic osteomyelitis is defined as long-standing infection that evolves over months or even years, characterized by the persistence of microorganisms, low-grade inflammation, and the presence of dead bone (sequestrum) and fistulous tracts [2, 4, 6, 7]. Subacute osteomyelitis is a form of osteomyelitis with insidious onset, which is difficult to diagnose because the characteristic signs and symptoms of the acute form of the disease are absent [8–11]. Subacute osteomyelitis may develop after a previous acute attack of osteomyelitis, but may also present in patients with no previous acute attack without having received antibiotics [12].

Brodie’s abscess, one type of subacute osteomyelitis, was first described in the literature in 1832 by Sir Benjamin Brodie, as a localized bone abscess that developed without prior systemic illness [13]. Wiles (1951) referred to Brodie’s abscess as a special form of chronic osteomyelitis, which follows an acute attack, when the virulence of the organism and the resistance of the patient are evenly balanced [12]. In 1965, Harris and Kirkaldy-Willis were the first to describe and publish a report on primary subacute osteomyelitis, which develops without previous acute attack [12]. In 1973, Gledhill developed a radiological classification for primary subacute osteomyelitis consisting of four types [14]. This system was later modified and expanded by Roberts *et al.* into six types in 1982 [15]. In both classifications, Type 1 represents the classic Brodie’s abscess [4].

Brodie’s abscess is defined as a centrally placed, sharply circumscribed bone abscess with a sclerotic wall. It represents 2.4 % of osteomyelitis cases [16], but in East Africa this form of osteomyelitis has been reported to be the most common [12]. *Staphylococcus aureus* is the most common affecting organism, followed by *Streptococcus* species [17, 18], but other organisms have also been reported [19]. Of the cases, 25 % are sterile [20, 21]. With a 3:2 ratio, boys are more commonly affected, and it usually occurs in young patients with an average age of 19.5 years [20]. The lower extremity is more commonly affected, with the tibia being the most common location [15], while occurrence in the upper extremity accounts for only approximately 9 % of cases [20, 21]. Metaphysis of the long bones is most commonly affected [12]. Diaphyseal involvement is reportedly more common in adults [11, 15, 16]. However, other sites such as the distal radius [21] and tarsal cuboid bone [22] have also been reported, although rarely.

The most common presenting symptom is mild to moderate pain of insidious onset that has been present for weeks or months, with insignificant or absent general reaction to the infection and minimum physical signs [12]. The patient is typically afebrile at presentation and their temperature is often normal throughout the illness [12]. Fever may also occur in cases of fulminant actively spreading septicemia [17, 23]. Localized tenderness and swelling are the only local physical signs, although one or both may be absent [12]. Painful movement of the adjacent joint with or without mild joint effusion may also be present. Impaired limb function, such as a limp, or severe wasting of the quadriceps muscle with loss of the patellar tendon reflex may also be present [24].

Laboratory investigations include erythrocyte sedimentation rate (ESR), C-reactive protein (CRP), white blood cell count (WBC) and blood culture. The total WBC is usually normal and the differential count is only slightly more reliable [12]. The ESR, although often normal, is more reliable than the WBC [12]. In some cases of staphylococcal infections, a very high reading, over 50 mm/hour, has been reported [12]. CRP may be normal or mildly elevated. A raised CRP and ESR together increase their sensitivity to nearly 98 % [25, 26]. The blood culture is usually negative, as the patients do not have septicemia [12]. Staphylococcal antibody titers provide another test of a diagnostic value [12], as described by Lack and Towers in 1962 [27]. Lack and Towers measured antibodies to alpha-hemolysin, leukocidin, staphylocoagulase and to staphylokinase in the sera of patients with osteomyelitis. Serial determinations were then made at regular intervals for at least 6 months. By the methods used, anti-leukocidin levels rose more constantly than anti-alpha-hemolysin; anti-coagulase levels rose and fell more slowly and were of less help diagnostically. Anti-staphylokinase was often the first to rise but was less constant. Of the confirmed cases of osteomyelitis, 78 % showed elevated anti-alpha-hemolysin titers, 70 % showed raised anti-leukocidin titers and approximately 50 % showed raised anti-coagulase titers. It has therefore been suggested that at least two antibodies should be measured as a diagnostic aid and that more attention should be paid to rising titers than to single estimation. In 1969, King and Mayo conducted a study based on a survey of 67 patients with subacute hematogenous osteomyelitis, where the nature of the disease, the clinical types of infection and the treatment were described. As a part of the investigations the anti-staphylolysin titer was estimated in 21 cases. Their findings, however, did not support the findings of Lack and Towers [27], where the anti-staphylolysin-titer did not appear to be a very useful test, as only 7 of the 21 estimations made were positive [11].

Imaging studies include plain radiographs, Technetium-99 bone scintigraphy, ultrasound and magnetic resonance imaging (MRI) [26]. MRI is the most effective imaging technique for diagnosing osteomyelitis [26]. It has been shown to be very sensitive and, in some cases, specific for the detection and staging of osteomyelitis [28]. MRI in Brodie’s abscess helps to delineate the extent of infection, usually indicating a well-circumscribed lesion without the more widespread involvement typical of diffuse osteomyelitis [21, 29]. The MRI appearance of Brodie’s abscess, the ‘target’ appearance, with a center two rings and a peripheral halo was first described by Martí-Bonmatí *et al.* [30].

Important differential diagnoses of Brodie’s abscess include unicameral bone cyst, osteoblastoma, fracture, osteosarcoma, aneurysmal bone cyst, Ewing’s sarcoma, osteoid osteoma, reticulosarcoma, multiple myeloma, giant cell tumor, acute hematogenous osteomyelitis and chronic osteomyelitis [23].

Treatment of Brodie’s abscess ranges from curettage, biopsy and culture, the use of impregnated antibiotic beads, cancellous bone grafting after curettage followed by immobilization to antibiotics alone without surgery [16]. It has been suggested that antibiotics alone are sufficient for the treatment of subacute osteomyelitis, and that surgery should be reserved for aggressive lesions [24] with ESR of greater than 40 ml/hour, abscess >3 cm or a lesion indistinguishable from a tumor [16]. Olasinde *et al.* also reported satisfactory outcomes in all patients who underwent curettage, biopsy for histology and culture, as well as cancellous bone grafting and postoperative antibiotic course, with no recurrence at a minimum follow-up of 2 years and complete obliteration of the abscess cavities and development of normal trabeculae bone pattern [16]. In general, 6 weeks of antibiotics administrated wholly or partially by an intravenous route is recommended [31]. However, a total of 6 weeks of antibiotic treatment may be sufficient in uncomplicated cases [26].

## Case presentation

A 17-year-old white boy presented to our out-patient clinic with a 6-month history of pain in his left knee joint. His complaint had an insidious onset and his pain was mostly upon weight bearing. No giving-way was reported: giving-way is a complaint by the patient about losing single leg stance because the joint subluxes due to pathological laxity, which is a symptom of joint instability. During the 6 months of his complaint he did not report any swelling or redness of his left knee or leg. Fever was also absent. Oral painkillers did not produce significant pain relief. There was no history of trauma to the extremity. His family history was insignificant without any diseases. He had not undergone any operations in the past and did not take any regular medications. He had no known allergies.

He was afebrile at presentation. A physical examination revealed no gait disturbances; he walked normally without any limping. By inspection, following the exposure of his lower limbs bilaterally, no scars, redness, rashes, muscle wasting, obvious swelling or obvious deformity (varus, bow-legged; or valgus, knock-knees) were seen. On palpating his knee joint there was no warmth. His skin temperature was bilaterally normal and similar. No joint effusion was felt. A patella tap test was negative. The patella tap test is a test to detect joint effusion, where the examiner, with extended knee of the patient, applies pressure with one hand above the knee to empty the suprapatellar pouch into the knee joint. With the other hand the patella will be tapped with a downward force. A positive test is if the patella can be felt touching (tapping) the femur*.* There were no retropatellar pains or crepitation. Zohlen sign was negative. In the Zohlen test, the patient’s knee is extended, and the examiner gently presses the patella into the trochlear groove while asking the patient to tense the extensor muscles of the thigh: quadriceps femoris. If this manoeuver causes pain, the test is positive and it is a sign of femoropatellar joint irritation*.* There was slight tenderness at his medial joint space and medial tibia plateau, but no tenderness at the border of his patella, lateral joint space or patellar tendon insertion on palpation. No palpable Baker’s cyst in the popliteal fossa could be detected. The range of motion of his left knee joint was, according to the Neutral-0 Method, extension/flexion 0-0-125 degrees. Pain was produced on maximal knee flexion. His anterior cruciate ligament (ACL) was stable, with negative ‘anterior drawer’ and ‘Lachman’s’ tests: the Lachman’s test is a variant of the anterior drawer test, and is performed between 20 and 30 degrees of knee flexion to test for the stability of the anterior translation of the knee provided by the ACL. His ‘pivot shift’ test was also negative. The pivot shift test is another test that can accurately indicate an ACL rupture. The test is performed with the patient’s knee starting in full extension. While maintaining internal rotation of the tibia, a valgus force is applied and the knee is slowly flexed to 25 and 30 degrees. The examiner will feel for a subluxation of the lateral tibial plateau as it reduces to its normal position, which indicates an ACL rupture. His posterior cruciate ligament (PCL) was stable, with negative ‘posterior drawer’ and ‘quadriceps’ tests. The quadriceps test is used to diagnose PCL rupture and to measure posterior laxity of the knee. The test is performed with the knee flexed between 80 and 90 degrees and in neutral rotation. From the initial position, the patient is asked to fire the quadriceps muscle while the examiner applies counter pressure against the ankle. As the quadriceps applies pressure, any posterior translation of the tibia on the femur will be reduced back to a normal position*.* Varus (medial) stress and valgus (lateral) stress tests (in 0 and 15 degrees of flexion) were negative indicating stable lateral and medial collateral ligaments, respectively. McMurray’s and Steinmann 1 tests were positive for the medial meniscus. McMurray and Steinmann 1 are tests to diagnose meniscal pathology. The tests are performed with the hip and knee at 90 degrees of flexion. The tibia is rotated medially and laterally. The test is positive if lateral pain is elicited on medial rotation and medial pain is elicited on lateral rotation. The tests are performed at various degrees of knee flexion.

**Fig. 1:**
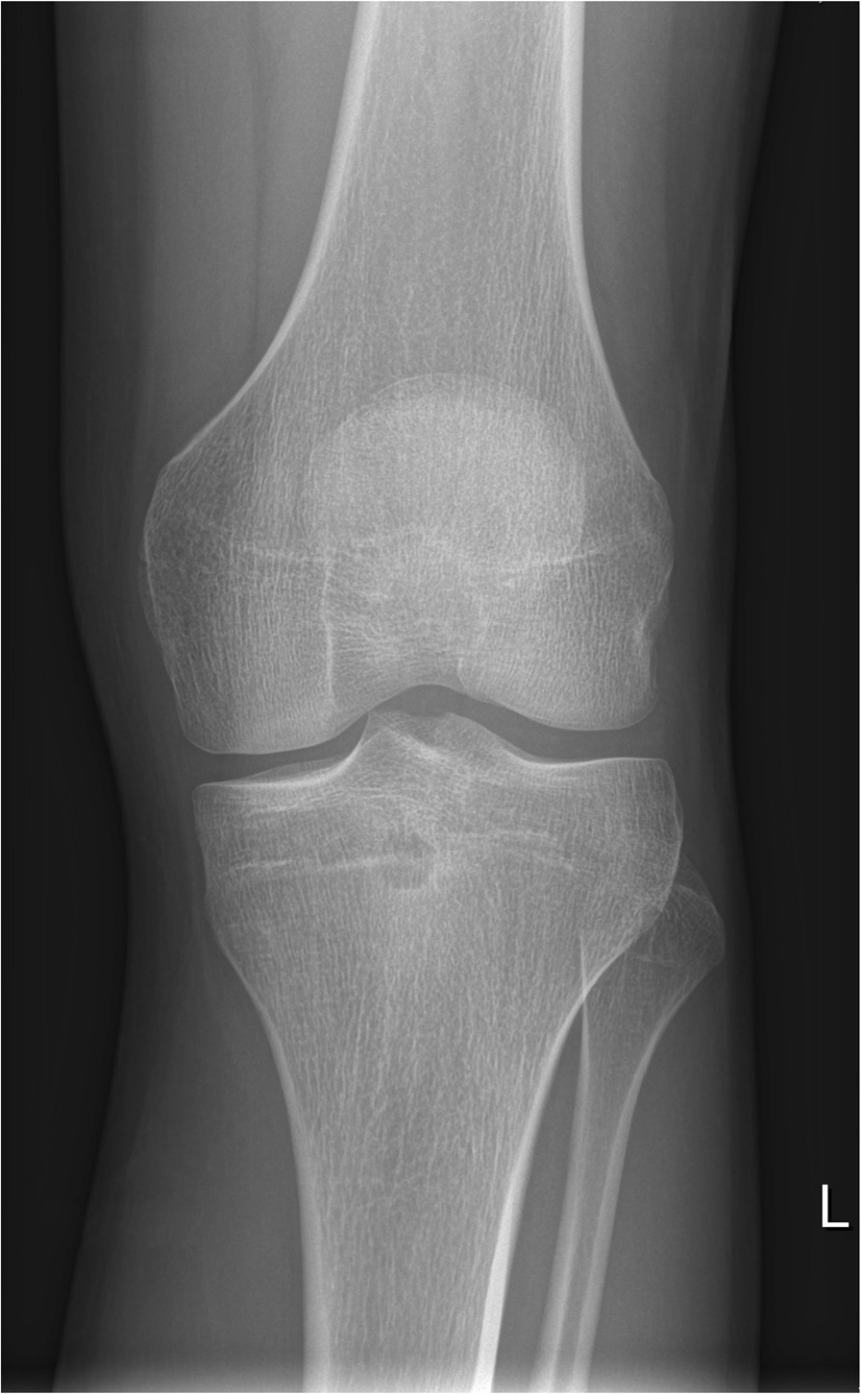
X-ray of the left knee (anteroposterior view), taken at the first out-patient presentation

**Fig. 2:**
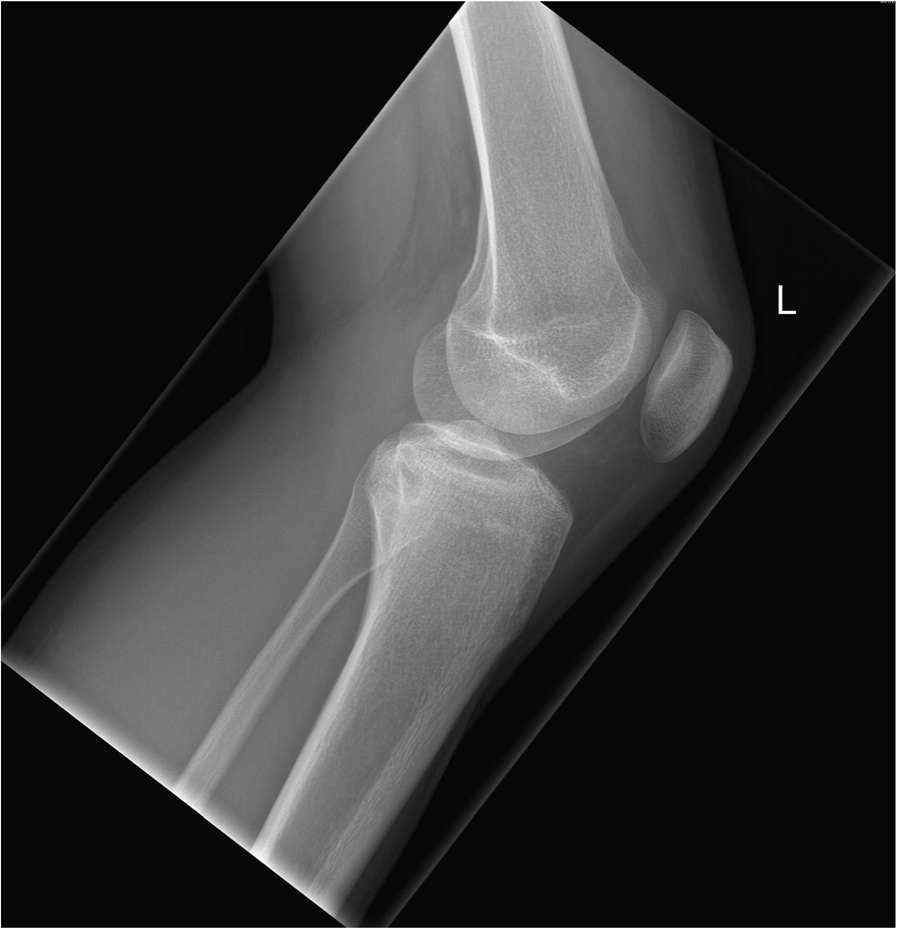
X-ray of the left knee (lateral view), taken at the first out-patient presentation

Anteroposterior and lateral X-ray of his left knee joint showed a small rounded radiolucent lesion in his proximal tibia, with no periosteal reaction (Figs. 1 and 2), which was interpreted as a simple bone cyst. Otherwise the X-ray was normal. With clinically absent infection signs, we did not take any blood samples or perform any laboratory tests. With the suspected diagnosis of medial meniscus damage, to identify the nature of the bone cyst and to exclude the presence of a tumor, we scheduled for him a regular appointment 2 weeks later for an MRI of his left knee in our radiology department. We prescribed him further oral painkillers in the interim.

**Fig. 3:**
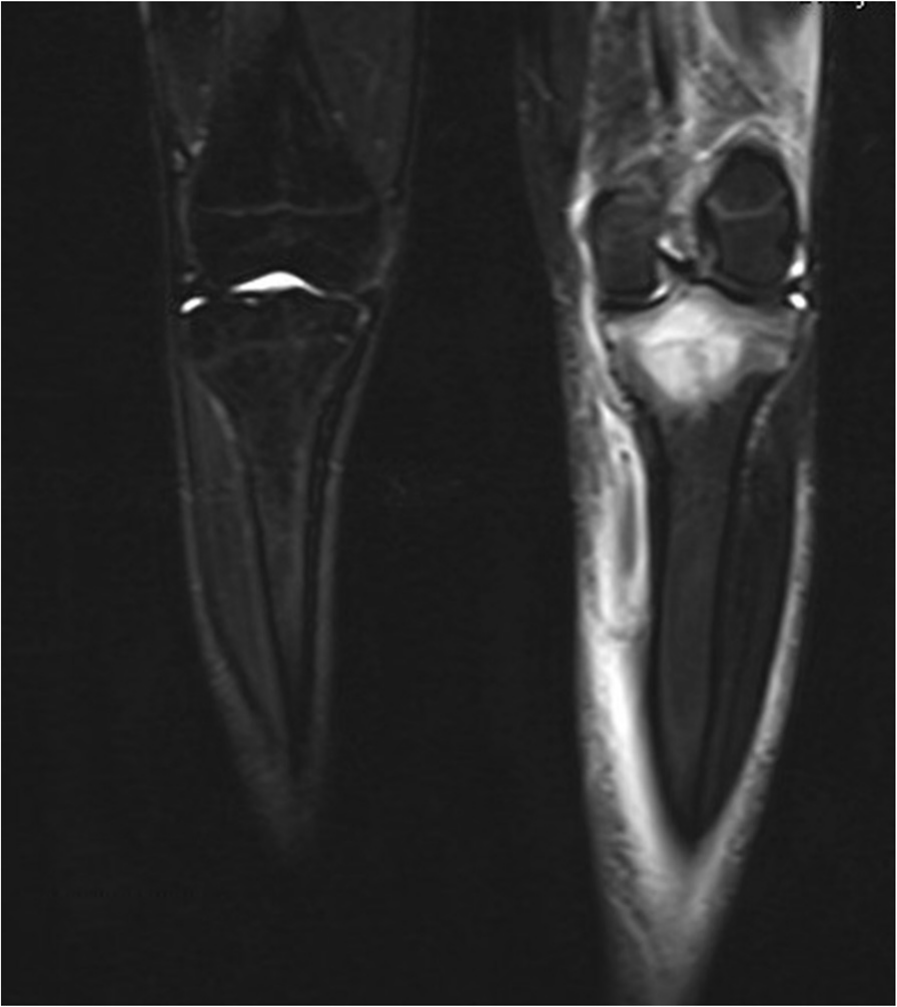
Magnetic resonance imaging scan of the left knee, taken at the day of the acute presentation, showing the intramedullary cystic lesion

**Fig. 4:**
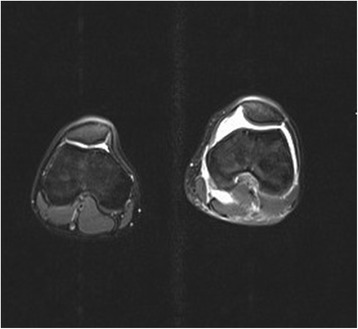
Magnetic resonance imaging scan of the left knee, taken at the day of the acute presentation, showing suprapatellar joint effusion

**Fig. 5:**
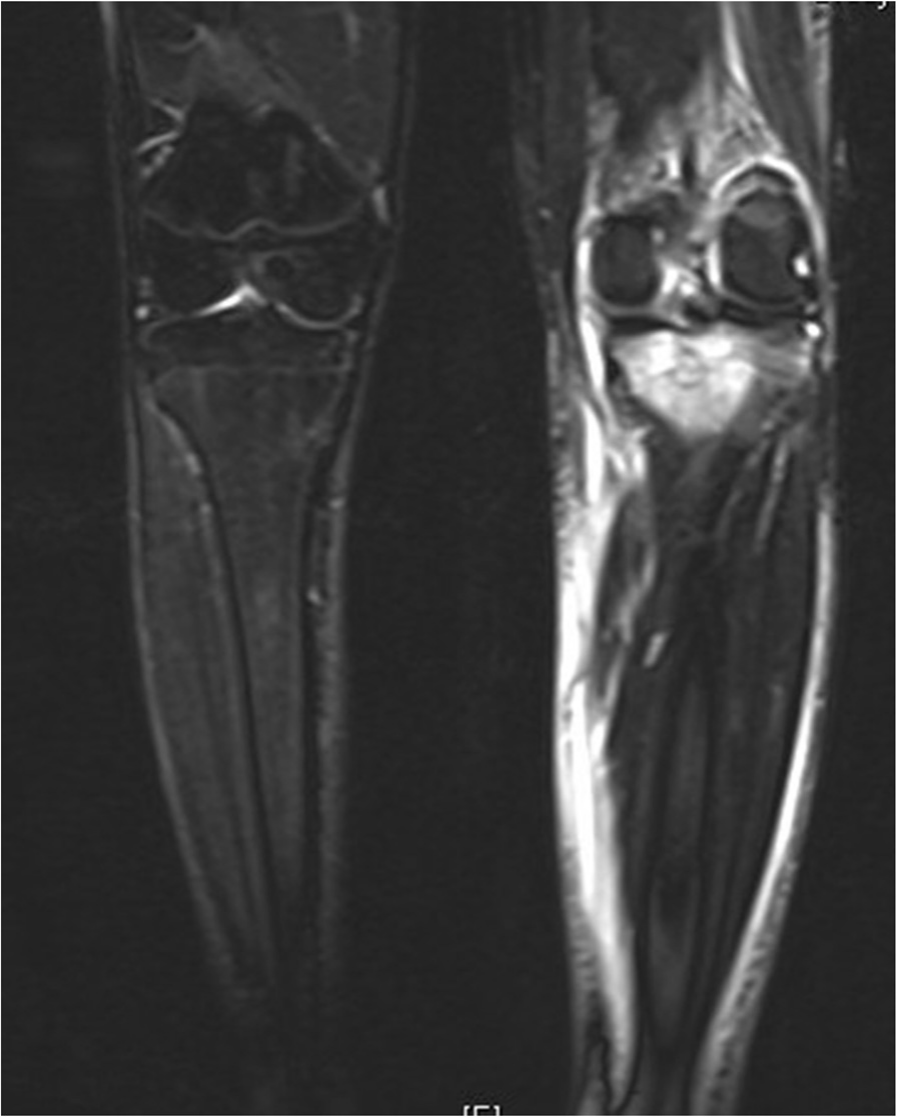
Magnetic resonance imaging scan of the left knee, taken at the day of the acute presentation, showing the leg fasciitis and myositis

One week after his initial presentation to us, he presented acutely in our emergency department complaining of a sudden increase in the pain and swelling of his knee joint, with pain and swelling of his left leg. No history of a recent trauma was reported. A physical examination revealed swelling and warmth of his left knee joint with maximal tenderness in the dorsal aspect of the joint. Swelling, tenderness and warmth of his left calf were also noted. Laboratory values at the time of admission showed elevated CRP of 60.8 mg/l, normal leukocyte count of 9.3 and elevated D-dimer of 1.22 mg/l. An ultrasound of his left knee and Doppler ultrasound of his left leg were performed. A deep vein thrombosis (DVT) could be excluded. An incarcerated Baker’s cyst with suspected rupture or bleeding in the cyst was reported. An MRI of his left knee and leg was recommended. This was performed on the same day to further clarify the diagnosis and exclude leg compartment syndrome. The MRI showed a well-defined central intramedullary cystic lesion with fluid collection in the proximal tibia metaphysis extending to the epiphysis with extensive surrounding reactive bone marrow edema (Fig. 3). Extensive suprapatellar joint effusion with reactive synovitis was also seen (Fig. 4). Partial fasciitis of his left leg and myositis of the medial head of his gastrocnemius muscle were also described (Fig. 5). The MRI finding was suggestive of a Brodie’s abscess. The synovial fluid of his left knee was aspirated and was sent to microbiological examination, where no bacteria were isolated.

Emergency surgery was indicated and performed on the same day. Open surgical debridement and curettage with drainage of the abscess through a posterior approach was performed. Putrid turbid fluid was evacuated from the lesion and sent for bacteriological examination and culture. Cultures were positive for *Staphylococcus aureus.* Histologic tissue examination showed evidence of focal osteomyelitis without signs of malignancy. The patient’s left knee was immobilized for 2 weeks until complete wound healing, using a splint (Mecron-Schiene; DARCO, Europe, GmbH, Raisting, Germany). He was postoperatively treated with antibiotics (flucloxacillin), initially intravenously and then orally, for an overall period of 5 weeks (2 weeks until discharge and then a further 3 weeks). Under antibiotics his CRP decreased so that on discharge he had a normal CRP of 1.3 mg/l and a normal leukocyte count of 5.83. He was discharged 2 weeks postoperatively. He was advised to be non-weight-bearing for 3 weeks postoperatively and then allowed 15 kg of weight bearing for a further 3 weeks. Full weight bearing was permitted 6 weeks postoperatively. Adequate thrombosis prophylaxis was performed during his in-patient stay and advised to be continued until full weight bearing was possible. We arranged a follow-up MRI appointment in our institution and an appointment in our out-patient clinic for a follow-up clinical examination 3 months postoperatively.

**Fig. 6:**
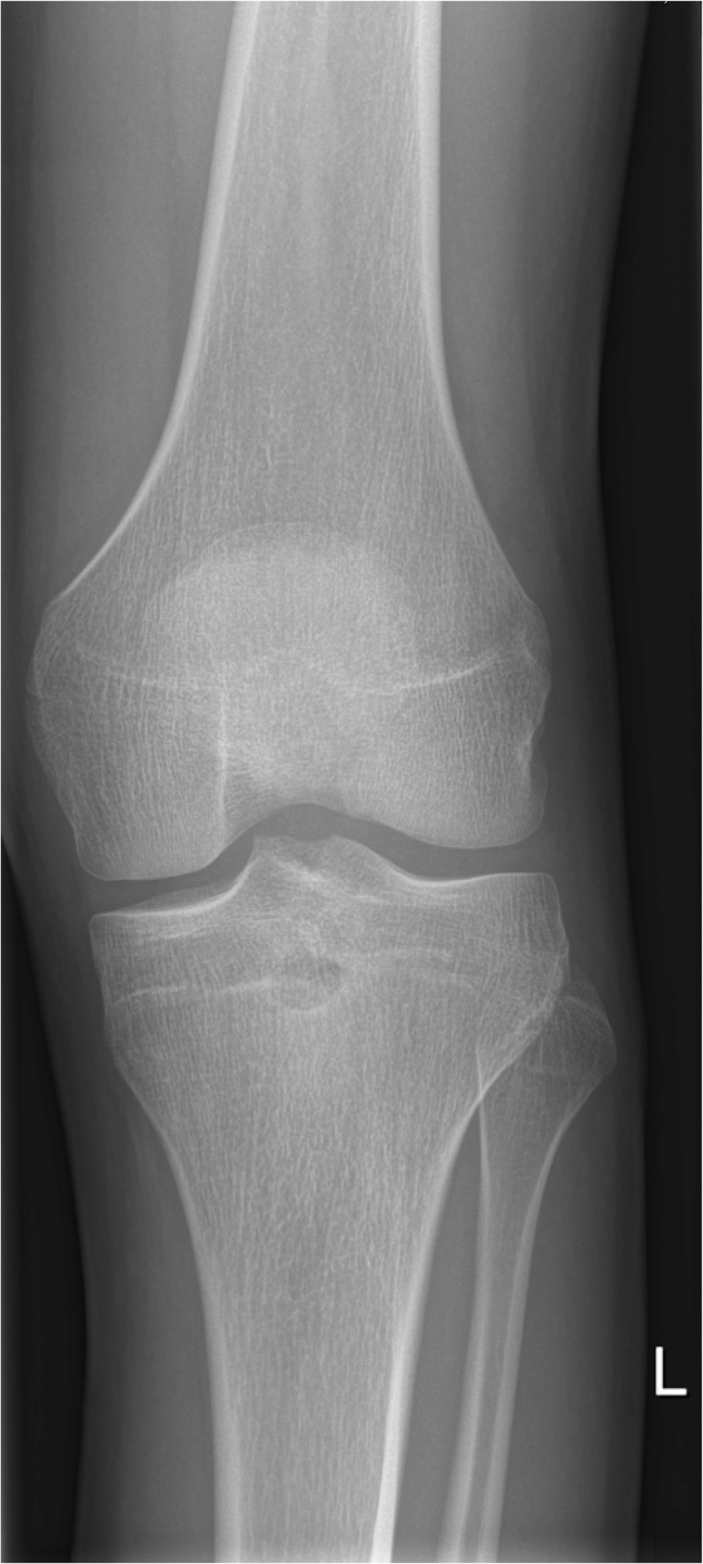
X-ray of the left knee (anteroposterior view), taken at the first postoperative out-patient presentation (10 weeks postoperative)

**Fig. 7:**
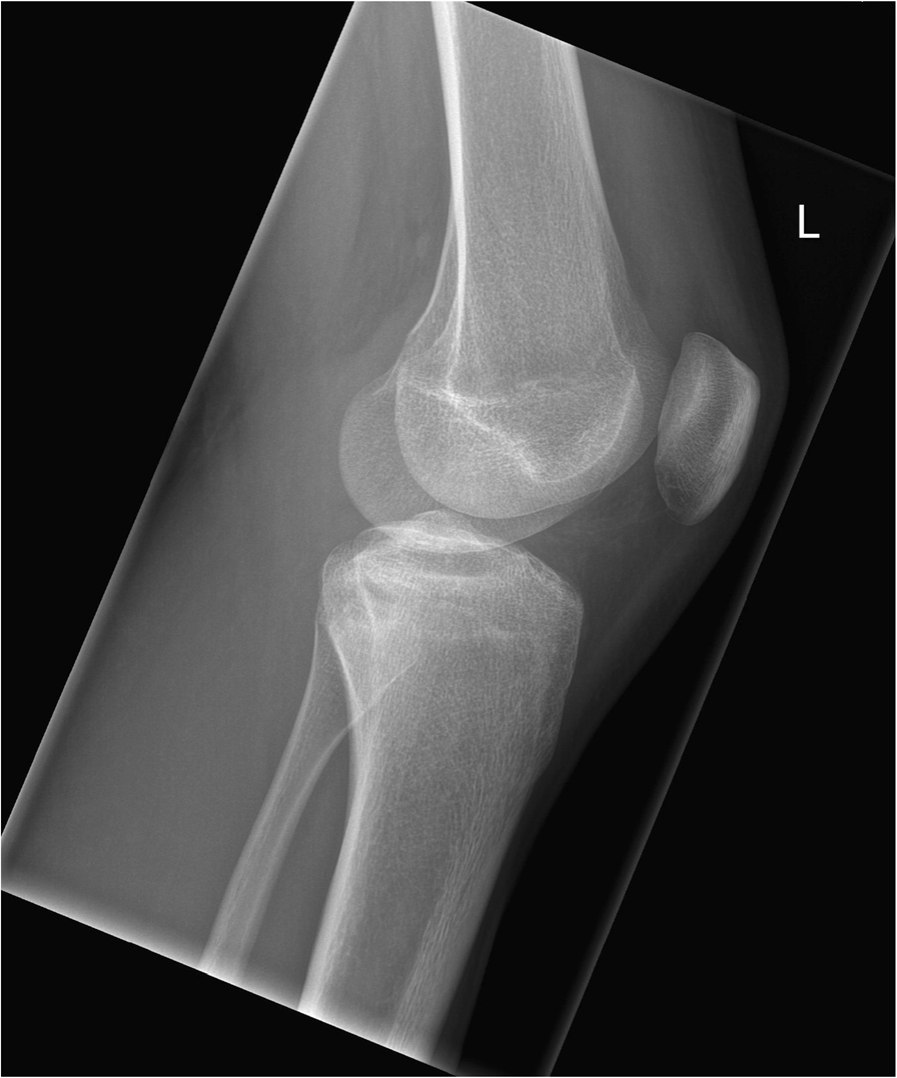
X-ray of the left knee (lateral view), taken at the first postoperative out-patient presentation (10 weeks postoperative)

However, he presented to our out-patient clinic 2 months postoperatively complaining of pain in his left knee following a direct fall on his knee that occurred 1 week previously. On physical examination there was no redness, warmth or an obvious swelling of his left knee. No joint effusion was felt. No local tenderness could be elicited. The active and passive movement of his left knee joint was free with normal range of motion. A plain X-ray of his left knee was performed, which did not show any fresh bone injury. A small radiolucent area in his proximal tibia was still seen (Figs. 6 and 7). He had a normal CRP of 0.3 mg/l and a normal leukocyte count of 8.01. He was reassured and advised to present 1 month later for the already arranged MRI and clinical follow-up appointments.

**Fig. 8:**
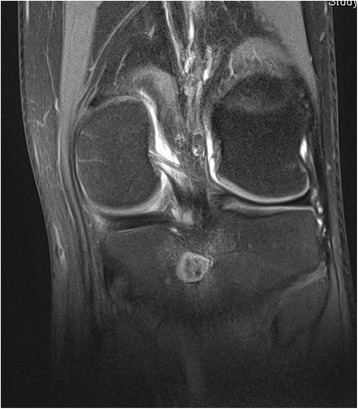
Magnetic resonance imaging scan of the left knee (coronal view), follow-up examination (3 months postoperative)

**Fig. 9:**
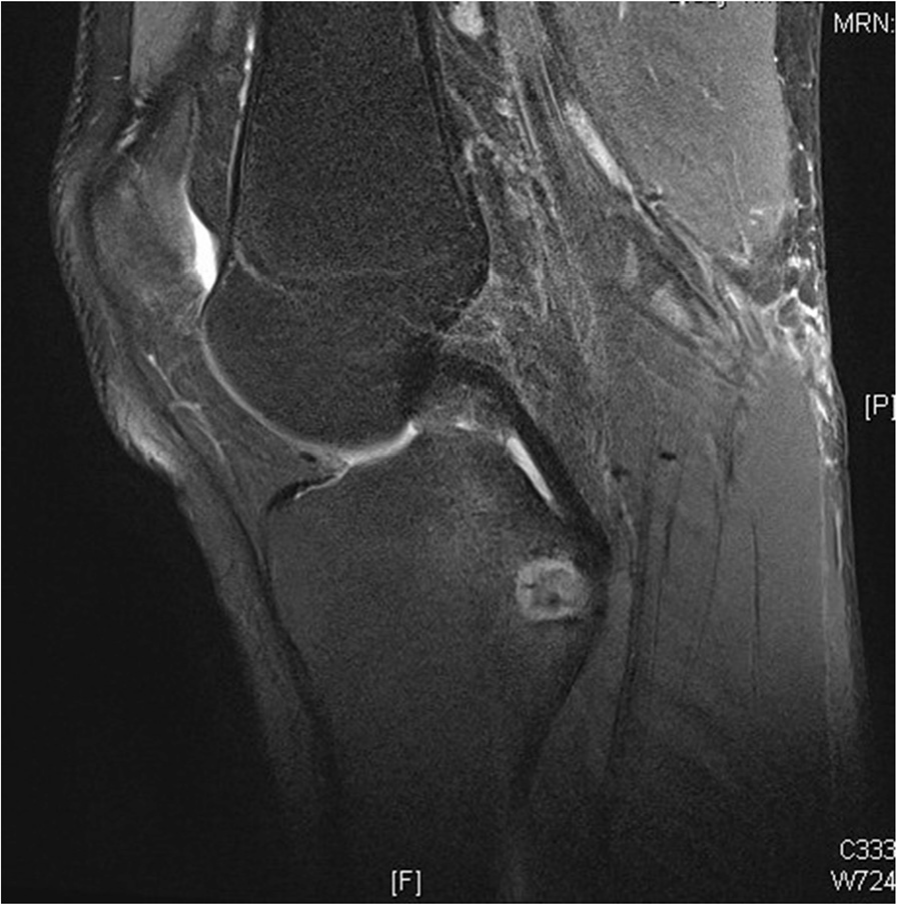
Magnetic resonance imaging scan of the left knee (sagittal view), follow-up examination (3 months postoperative)

One month later (3 months postoperatively) a follow-up MRI of his left knee was performed; he then presented to our out-patient clinic for a follow-up clinical examination. The MRI revealed a residual defect in his proximal tibia with a little residual reactive bone marrow edema with no joint effusion, no signs of synovitis, inflammation or abscess formation (Figs. 8 and 9). He was asymptomatic and pain-free, with no residual local tenderness. A follow-up examination in our out-patient clinic in 6 months was recommended.

## Discussion

An online search of PubMed using the keyword ‘Brodie’s abscess’ revealed 116 studies, of which 70 were excluded because of irrelevance or lacking the complete required data. The remaining 46 studies were thoroughly reviewed to identify clinical presentation similar to our case.

Acute pain onset and systemic reaction in Brodie’s abscess have rarely been reported. A study with a total of 1037 patients with osteomyelitis was performed, where all patients had pain and local swelling but none had fever, localized tenderness or warmth [16]. An acute increase in pain severity following a fall on the affected extremity has been reported [32]. In our case the patient presented to us initially with pain of insidious onset that had been present for several months. What makes our case unusual is the acute sudden increase in his knee pain and swelling, and his acute leg pain with calf tenderness and warmth, with elevated CRP of 60.8 mg/l and elevated D-dimer of 1.22 mg/l, without any precipitating event or trauma. Because of this acute presentation, which is atypical for Brodie’s abscess, and which did happen because of the resulting fasciitis and myositis, it was initially confusing to consider Brodie’s abscess as a differential diagnosis. The two initial differential diagnoses postulated were DVT and suspected compartment of the leg by ruptured Baker’s cyst. Because of his calf warmth and tenderness, and his elevated D-dimer, DVT was suspected, which was easily excluded with Doppler ultrasound. Because of his leg swelling and tenderness, the elevated CRP and the diagnosed Baker’s cyst in ultrasound, an incarcerated Baker’s cyst with ruptured cyst or bleeding in the cyst with suspected resulting compartment of the leg was our second differential diagnosis. This was clarified as soon as the MRI was performed.

## Conclusions

Brodie’s abscess is an uncommon form of osteomyelitis, which is difficult to diagnose in the early stages because of the insidious onset of symptoms, lack of systemic response and in most cases normal laboratory values. The most common symptom is mild to moderate pain of insidious onset that has been present for months. However, sudden acute exacerbation of the complaint with swelling and tenderness of the whole affected extremity, without any trauma or other precipitating factors, may occur as a result of resulting fasciitis or myositis of the affected extremity. Therefore in cases of acute increase in joint or extremity pain or swelling that has been insidiously present for months, Brodie’s abscess should be kept in mind as one of the differential diagnoses, as it may present acutely in cases with accompanying fasciitis and myositis, and may be clinically mistaken for DVT or ruptured Baker’s cyst with resulting compartment, in cases where the lower extremity is affected. MRI remains the gold standard imaging study and it has been shown to be very sensitive in the diagnosis of Brodie’s abscess. Surgical curettage, biopsy for culture and histology and a postoperative antibiotic course (initially intravenous and then orally) remains from our point of view the standard treatment, and according to our experience would be further recommended.

## Consent

Written informed consent was obtained from the patient and his mother for publication of this case report and any accompanying images. A copy of the written consent is available for review by the Editor-in-Chief of this journal.

## Abbreviations

ACL: anterior cruciate ligament
CRP: C-reactive protein
DVT: deep vein thrombosis
ESR: erythrocyte sedimentation rate
MRI: magnetic resonance imaging
PCL: posterior cruciate ligament
WBC: white blood cell count
